# Tracking Pertussis and Evaluating Control Measures through Enhanced Pertussis Surveillance, Emerging Infections Program, United States

**DOI:** 10.3201/eid2109.150023

**Published:** 2015-09

**Authors:** Tami H. Skoff, Joan Baumbach, Paul R. Cieslak

**Affiliations:** Centers for Disease Control and Prevention, Atlanta, Georgia, USA (T.H. Skoff);; New Mexico Department of Health, Santa Fe, New Mexico, USA (J. Baumbach);; Oregon Health Authority, Portland, Oregon, USA (P.R. Cieslak)

**Keywords:** pertussis, whooping cough, Bordetella pertussis, bacteria, surveillance, preventable bacterial disease, vaccine, National Notifiable Disease Surveillance System, Enhanced Pertussis Surveillance, Emerging Infections Program, EIP

## Abstract

Despite high coverage with pertussis-containing vaccines, pertussis remains endemic to the United States. There have been increases in reported cases in recent years, punctuated by striking epidemics and shifting epidemiology, both of which raise questions about current policies regarding its prevention and control. Limited data on pertussis reported through the National Notifiable Disease Surveillance System have proved insufficient to answer these questions. To address shortcomings of national pertussis data, the Emerging Infections Program at the US Centers for Disease Control and Prevention launched Enhanced Pertussis Surveillance (EPS), which is characterized by systematic case ascertainment, augmented data collection, and collection of *Bordetella pertussis* isolates. Data collected through EPS have been instrumental in understanding the rapidly evolving epidemiology and molecular epidemiology of pertussis and have contributed essential information regarding pertussis vaccines. EPS also serves as a platform for conducting critical and timely evaluations of pertussis prevention and control strategies, including targeting of vaccinations and antimicrobial prophylaxis.

## Abstract 2

This network can improve pertussis prevention and control and be a model for surveillance programs.

Pertussis (whooping cough) has proven to be a frustratingly persistent public health problem. Although annual numbers of reported cases decreased >99% in the United States after introduction of whole-cell pertussis vaccines in the 1940s, this highly contagious respiratory illness has refused to go the way of other vaccine-preventable diseases of childhood, such as polio, *Haemophilus influenzae* type b infection, and diphtheria. Pertussis remains endemic to the United States, and the number of reported cases has been increasing steadily since the late 1980s, with notable epidemic peaks in recent years (Figure 1). In 2012, more than 48,000 cases were reported nationally, the largest number since 1955. Possible reasons for the observed increase include changes in diagnostic testing and reporting, increased provider and public awareness, mismatch of vaccine antigens and circulating strains, and reduced duration of immunity from acellular pertussis (aP) vaccines that replaced whole-cell vaccines in the United States during the 1990s.

**Figure 1:**
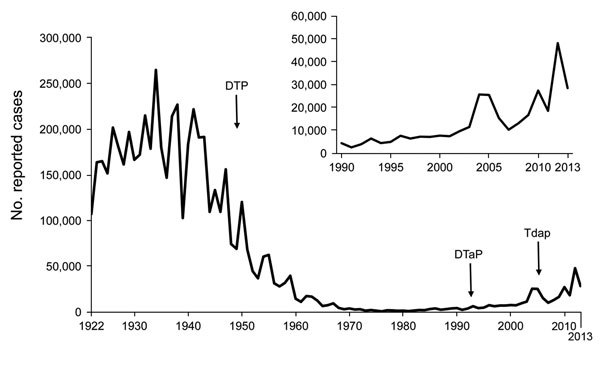
Reported pertussis cases from the National Notifiable Diseases Surveillance System, United States, 1922–2013. Inset show cases during 1990–2013. Data for 1950–2013 were obtained from the Centers for Disease Control and Prevention National Notifiable Diseases Surveillance System and Supplemental Pertussis Surveillance System. Data for 1922–1949 were obtained from passive reports to the US Public Health Service. DTP, diphtheria and tetanus toxoids combined with whole-cell pertussis vaccine; DTaP, diphtheria and tetanus toxoids and acellular pertussis vaccine; Tdap, reduced-dose acellular pertussis vaccine combined with tetanus and diphtheria toxoids.

The cough illness associated with pertussis can be quite severe and the disease debilitating in persons of all ages, but illness and death rates remain highest among young infants, especially those too young to be directly protected by vaccination. Recently, the epidemiology of pertussis has indicated an increasing burden of disease among school-age children and adolescents, most of whom are up-to-date on pertussis vaccinations (*1**,**2*). Changes have also been identified in *Bordetella pertussis* at the molecular level, such as loss of pertactin, a key aP vaccine antigen (*3*).

Pertussis has been a reportable disease in the United States since 1922. Case-based surveillance data are captured through the National Notifiable Diseases Surveillance System (NNDSS) from 57 public health jurisdictions (50 states; 5 US territories; New York, NY; and Washington, DC) (*4*). NNDSS is a passive system that relies on reports from health care providers and laboratories, probably resulting in underreporting of cases. In addition, because case investigation requires the effort and resources of disparate local and state public health agencies, the quantity and quality of pertussis case reports vary, and data elements fundamental to the understanding of pertussis, including case demographics, clinical symptoms and pertussis vaccination history, are often incomplete. NNDSS is a relatively inflexible system that cannot readily accommodate newly desired data elements, and complex data transmission processes and challenges might compromise the quality of data received at the Centers for Disease Control and Prevention (CDC).

Although NNDSS has been essential for monitoring the national burden of pertussis and age-related trends in disease over time, data are of insufficient detail and consistency to answer reliably the many urgent questions relevant to public health. Are current pertussis prevention and control strategies effective, specifically, vaccination and postexposure antimicrobial chemoprophylaxis (PEP)? Has the spectrum of clinical illness changed, and does it differ by factors such as age and vaccination status? In the setting of waning aP-induced immunity, should additional doses of the tetanus toxoid, reduced diphtheria toxoid, and acellular pertussis vaccine (Tdap) be recommended, and if so, for which populations? Is *B. pertussis* evolving in key ways at the molecular level, and what, if any, is the clinical and epidemiologic relevance of identified changes? What are the disease burden and epidemiologic and molecular characteristics of other *Bordetella* species, and how might these species be contributing to the resurgence of pertussis-like cough illness?

## Enhanced Pertussis Surveillance System

In 2011, Enhanced Pertussis Surveillance (EPS) was undertaken by 6 states within the Emerging Infections Program (EIP), a collaborative network between CDC and state and local health departments, academic institutions and laboratories that serves as a national resource for surveillance, prevention, and control of emerging infectious diseases (*5*). EPS was initiated in EIP sites that had varying levels of *B. pertussis* incidence and existing pertussis surveillance infrastructure. The principal objectives of EPS are to determine overall and age-specific incidence and epidemiologic characteristics of pertussis, to characterize the molecular epidemiology of circulating *B. pertussis* strains, to monitor the effects of pertussis vaccines, and to provide a platform for conducting special studies, including critical and timely evaluations of pertussis prevention and control strategies. As a secondary objective, the system collects data to describe the epidemiology and molecular characteristics of other *Bordetella* species, including *B. holmseii*, *B. parapertussis*, and *B. bronchiseptica*.

For efficiency, EPS was built upon the NNDSS pertussis surveillance infrastructure within participating states, leveraging and enhancing existing efforts; within the same catchment area, cases reported through EPS are also reported through NNDSS. As with NNDSS, case investigations are triggered by a positive pertussis laboratory result or report from a diagnosing health care provider, and follow-up is initiated by the local public health system. EPS cases are classified according to the NNDSS/Council of State and Territorial Epidemiologists (CSTE) pertussis case definition, and all modifications made to the case definition at the national level are adopted by EPS (*6*). Similar to NNDSS, EPS is population-based, thereby maximizing the generalizability of its findings.

Although NNDSS serves as a foundation for EPS, a substantial investment of resources is made to EPS states annually, and additional personnel are employed to conduct a higher-level of pertussis surveillance that is sustainable in the longer term. The specific enhancements of EPS involve the following items.

### Optimizing Case Detection and Reporting and Ensuring Consistency across Sites

As resources permit, EPS sites educate and encourage area health care providers, including pediatricians, internists, and family practitioners, to consider pertussis as part of the differential diagnosis and to test for it properly. In some EPS sites, state public health laboratories offer pertussis testing (e.g., culture and real-time PCR) at no cost to catchment-area health care providers or to patients without access to health care to ensure testing whenever *B. pertussis* is suspected as a cause of illness.

### Expansion of Variables Collected

The standardized EPS case report form mirrors the NNDSS form, but collects several supplemental demographic, clinical, and epidemiologic variables. The EPS case report form is revised annually, maintaining the flexibility to address key public health questions in a timely manner.

### Aggressive Attempts to Capture Complete Case Report Form Data

Local investigators and surveillance personnel work to interview each case-patient or parent proxy and the case-patient’s diagnosing health care provider and complete follow-up interviews when necessary. Multiple procedures are used to obtain accurate vaccination histories, including routine review of state immunization information systems and school immunization records, and occasionally contacting additional health care providers of a case-patient.

### Site-Specific Strategies to Maximize Acquisition of Isolates from Case-Patients

This feature is an arduous task, given the increasing reliance on non–culture-based methods for diagnosis of infection with *B. pertussis*. Approaches range from promoting centralized testing at a state public health laboratory, to identification of sentinel site providers for specimen collection, to recovering isolates from PCR-positive specimens. Once collected, *B. pertussis* isolates are sent to CDC, where they undergo susceptibility testing to erythromycin and azithromycin and a full panel of molecular characterization, including pulsed-field gel electrophoresis, multilocus variable number tandem repeat analysis, and multilocus sequence typing. More recently, laboratory testing has evolved to include phenotypic and genotypic assays for detection of pertactin-deficient isolates, as well as whole-genome sequencing of *B. pertussis*.

### Expansion of Activities to Include Collection of *B. pertussis* Clinical Specimens

Key advancements have been made in molecular characterization of clinical isolates. In response to these advancements, EPS is positioned to monitor characteristics of a larger population of circulating strains and to follow the molecular epidemiology of pertussis.

As of 2014, EPS is conducted in the 5-county Denver metropolitan area of Colorado; 8 counties in metropolitan Atlanta, Georgia (added at the beginning of 2014); the 15-county Rochester and Albany areas of New York; the 3-county Portland area of Oregon; and statewide in Connecticut, Minnesota, and New Mexico. Although EPS is conducted in ≈5% of the US population, the demographic composition of the EPS catchment area is similar to the whole United States in terms of racial, ethnic, and age distributions, which enables characterization of the epidemiology of *B. pertussis* among select population groups.

## Accomplishments

Since its inception, data collected through the EPS system have maintained a higher level of completeness than surveillance data reported through NNDSS. A comparison of data collected from both systems during 2011–2012 found significantly more complete data from EPS on race (91% vs. 76%; p<0.001) and ethnicity (93% vs. 72%; p<0.001) (*7*). Dramatic differences in completeness have also been observed for key variables, such as cough onset date, duration of cough, hospitalization status, and pertussis vaccination history (Table). High-quality race and ethnicity data enabled an analysis of EPS data from Oregon that found higher rates of disease among Hispanic infants than non-Hispanic infants, and identified that household size, regardless of ethnicity, might be a key marker of increased exposure to pertussis (*8*). In addition, complete vaccination history served as the foundation of EPS analyses that have further demonstrated the correlation between severe disease and lack of vaccination, comparisons that would have been difficult to make with a high proportion of missing data (*9*).

**Table:** Completeness of pertussis surveillance data collected from the NNDSS and EPS, United States, 2011–2012*

Characteristic	Complete, %†		Difference, %
	NNDSS‡	EPS	
Race	76	91	15
Ethnicity	72	93	11
Any cough	79	100	21
Paroxysms	78	100	22
Whoop	74	97	23
Post-tussive vomiting	75	99	24
Primary symptoms known§	72	96	24
Cough onset date	66	100	34
Duration of cough	71	100	29
Hospitalized	73	99	26
≥1 vaccine date and type, age range 3 mo–7 y	71	99	28

*Data were obtained from Kamiya et al. (7). NNDSS, National Notifiable Disease Surveillance System; EPS, Enhanced Pertussis Surveillance. All p values for comparisons were <0.0001. †Unknown or missing responses were considered incomplete. ‡NNDSS completeness calculation excludes data from EPS area. §Cough, paroxysms, whoop, and post-tussive vomiting

**Figure 2:**
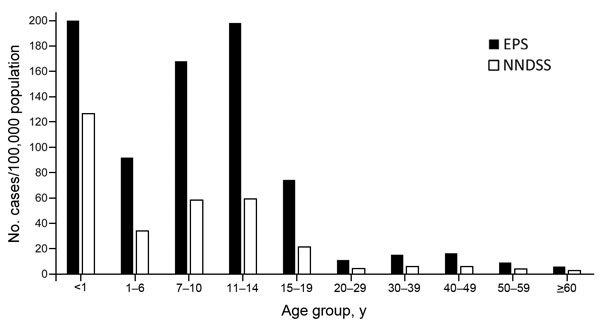
Overall and age-specific pertussis incidences, United States, 2012, from the National Notifiable Diseases Surveillance System (NNDSS) and Enhanced Pertussis Surveillance (EPS). Overall incidence for 2012. NNDSS: 15.4 cases/100,000 population (Centers for Disease Control and Prevention, NNDSS and Supplemental Pertussis Surveillance System, and 1922–1949 passive reports to the US Public Health Service). EPS: 42.0 cases/100,000 population (Emerging Infection Program, EPS for Colorado, Connecticut, Minnesota, New Mexico, New York, and Oregon).

Overall and age-specific incidence rates have tracked 1.5–3.3 times as high among EPS sites as national NNDSS rates (Figure 2). State-specific differences in pertussis incidence are recognized nationally, and states experience peaks at different times. Although differences between EPS and NNDSS certainly reflect variations in state-specific pertussis cycles and burden of disease, enhanced case ascertainment and awareness of the EPS program among diagnosing providers and local public health investigators also likely translates to increased case recognition and reporting within the EPS catchment area.

More than 20 EPS-specific data elements have been added to the case report form, many of which are intended to inform policy or help monitor the impact of new vaccine recommendations. As we await potential licensure of the expanded use of >1 dose of Tdap, and the Advisory Committee on Immunization Practices (ACIP) considers additional doses for special populations, EPS is tracking the burden of pertussis among health care personnel, a target group for which few data are available on the burden of pertussis. To protect young infants at highest risk for severe illness and death from pertussis, the ACIP recommended Tdap vaccination of pregnant women in October 2011 and expanded the recommendation in 2012 to include a dose during every pregnancy (*10*). Maternal Tdap vaccination history is being captured for all infant pertussis cases identified through EPS, along with timing of Tdap receipt in relation to pregnancy and reasons for not getting vaccinated during pregnancy. This information is being collected to determine the uptake of the recent vaccination recommendation and to identify any epidemiologic changes in infant disease.

Since 2011, EPS has been ascertaining pregnancy status for female case-patients; during 2011–2013, a total of 3.5% of case-patients 15–44 years of age were identified as being pregnant at the time of their pertussis infection (EPS, unpub. data). Little is known about the course of illness and complications of pertussis among pregnant women, a group for which pertussis vaccination is currently recommended as a means of protecting young infants. EPS has also been documenting the source of infant infection and has identified a shift from mothers to siblings as the most commonly identified source of disease transmission to infants (*11*). This finding is in contrast to those of previously published studies (*12**,**13*) in the United States and is crucial in the context of increasing burden of disease among school-age children.

EPS now serves as a key source of *B. pertussis* isolates for CDC, accounting for >50% of isolates received annually during 2011–2013. To date, >400 isolates have been collected from case-patients across the age spectrum; >80% of isolates have been obtained from case-patients >1 year of age. The availability of isolates linked to corresponding epidemiologic case data positions EPS to monitor the evolving molecular epidemiology of pertussis and quickly detect changes in the *B. pertussis* genome. EPS isolates were crucial to a recent analysis that identified emergence and rapid proliferation of pertactin-deficient strains in the United States (*3*). Isolates from EPS states conducting population-based surveillance over time helped illustrate the emergence of pertactin deficiency across the general population. Because of highly complete case report data, EPS data were also key to understanding the clinical and epidemiologic relevance of pertactin deficiency. Clinical symptom profiles were similar by pertactin status; however, vaccinated case-patients were more than 3 times as likely as unvaccinated case-patients to have pertactin-deficient isolates, suggesting a selective pressure of vaccination (*14*).

Another unique feature of the EPS system is its ability to influence national pertussis surveillance practices. Through the collection of ruled-out cases (i.e., PCR-confirmed cases that did not meet the 14-day cough requirement of the CSTE case definition), EPS gathered data that helped guide revisions to the national CSTE pertussis case definition for infants <1 year of age, which included removing the required 14-day cough for PCR-confirmed or epidemiologically linked cases (*6*). In addition, although serologic results are currently not considered confirmatory in the national case definition and the lack of standardization among the >40 commercially available assays in the United States makes interpretation of serologic results challenging, EPS has begun to investigate serologically confirmed cases to ensure consistency in identification of clinically compatible disease across sites. This activity should help to measure the additional burden of disease and workload resulting from routine investigation of serologically confirmed cases and lay the groundwork for future inclusion of serologic results into the CSTE case definition. EPS will also serve as a platform for piloting a revised case definition before it is implemented on a national level, a key step in this era of increased disease burden and limited resources.

## Special Studies Using the EPS Platform

One of the hallmarks of the EIP infrastructure is the flexibility to add special studies. The EPS platform has served as a foundation for several key pertussis projects ranging from resource-intensive, case–control evaluations to activities considered “low-hanging fruit.” Through EPS, it has been observed that ≈30% of pertussis hospitalizations are occurring in age groups other than infants and the elderly (EPS, unpub. data), prompting the question, why are older children and adults being hospitalized for pertussis? EPS investigators conduct expanded reviews of medical records of all hospitalized EPS case-patients. Data gathered enable characterization of the severity of infections in hospitalized patients across age groups, determination of reasons for hospital admission, documentation of underlying health conditions associated with severe illness, assessment of current practices in treatment, and outcomes of severe pertussis infection.

Although data suggested that maternal antibody transfer resulting from Tdap vaccination during pregnancy would probably confer protection and modify the severity of pertussis among infants, at the time the ACIP recommendation was made for women to receive a dose of Tdap during pregnancy, there was no direct evidence demonstrating effectiveness of the strategy in preventing infant disease (*15**–**17*). EIP has initiated a timely case–control evaluation of the new recommendation and will provide urgently needed data on the usefulness of the strategy in the United States, adding to the data available from the United Kingdom (*18**,**19*). In addition, the evaluation will include an assessment of older infants to identify any negative effects of maternally transferred pertussis antibodies on protection provided by the primary pertussis immunization series, a theoretical consequence and potential concern of vaccination during pregnancy.

In the setting of increasing pertussis burden and waning aP-induced immunity after pertussis vaccination, it is crucial to ensure the effectiveness of other strategies, such as administration of PEP to close contacts to support current prevention and control efforts. Secondary attack rates of pertussis are high within household settings, and data are limited on the effectiveness of newer macrolide antimicrobial drugs currently recommended for PEP after pertussis exposure. Selected EPS sites are embarking on a study to assess secondary transmission of *B. pertussis* among household contacts after a 5-day course of azithromycin PEP. This labor-intensive study requires identification of case-patient household contacts and follow-up and specimen collection at multiple time points. Results from this evaluation will aid in determining whether current PEP recommendations for household contacts are useful for preventing secondary transmission of disease and, being mindful of judicious antimicrobial drug use policies, will determine whether or not alternate PEP guidelines should be considered. In addition, the study will provide information on nasopharyngeal carriage of *B. pertussis* among asymptomatic household contacts before PEP, an area for which few data are available.

Before official establishment of EPS, the EIP infrastructure was used to evaluate the clinical accuracy of available pertussis diagnostics. Because PCR and serologic assays were being used more frequently to diagnose pertussis in the United States, a study looking at the clinical accuracy of current pertussis diagnostics was needed. Data collected from EIP sites during 2007–2011 are currently being used to estimate the clinical sensitivity, specificity, and predictive values of a CDC multiplex real-time PCR and a serologic assay (ELISA) developed by CDC and the Food and Drug Administration. In addition, the EIP sites are assessing the clinical utility of the tests as they relate to stage of pertussis illness, age of patient, antimicrobial drug use, and vaccination status. Data from the evaluation will ensure that validated, standardized laboratory assays are available to help improve the diagnosis and reporting of pertussis, which will ultimately facilitate prevention and control efforts.

## Future Opportunities for EIP

Current evidence indicates that the resurgence of pertussis in the United States is real and not simply an artifact of improved surveillance. Furthermore, current vaccination strategies are not expected to reduce further the growing burden of disease in the United States. Because pertussis remains a notable public health challenge, EPS is well-positioned to monitor the changing epidemiology of this disease and provide timely, reliable surveillance data to help answer key questions. The flexibility and expertise of the EIP network can be relied on to tackle challenging public health issues and to make direct recommendations to advance the prevention and control of pertussis in the United States and can serve as a model for collaborators abroad hoping to implement standardized surveillance for pertussis in the international setting.
